# First Report on Detection and Molecular Characterization of Astroviruses in Mongooses

**DOI:** 10.3390/v16081269

**Published:** 2024-08-08

**Authors:** Jessica L. Kulberg, Anne A. M. J. Becker, Yashpal S. Malik, Souvik Ghosh

**Affiliations:** 1 Department of Biomedical Sciences, Ross University School of Veterinary Medicine, Basseterre P.O. Box 334, Saint Kitts and Nevis; jessicakulberg@students.rossu.edu (J.L.K.); anne.amjbecker@gmail.com (A.A.M.J.B.); 2 ICAR-Indian Veterinary Research Institute, Mukteswar 263168, Uttarakhand, India; malikyps@gmail.com

**Keywords:** astroviruses, small Indian mongoose, RNA-dependent RNA polymerase

## Abstract

Applying a pan-astrovirus (AstV) RT-hemi-nested PCR assay, we report here high detection rates (28.3%, 15/53) of AstVs in the small Indian mongoose (*Urva auropunctata*) on the Caribbean Island of St. Kitts. Based on deduced amino acid (aa) identities and phylogenetic analysis of long RNA-dependent RNA polymerase (RdRp) sequences (~315 aa, partial RdRp), the AstVs detected in the mongooses (designated as Mon-AstVs) were classified into two distinct groups (deduced aa identities of 66.45–67.30% between the groups). The putative RdRps of the Mon-AstVs shared low deduced aa identities with those of AstVs from other host species (<69%, <54%, and <50% identities with reptilian/amphibian AstVs, avastroviruses, and mamastroviruses, respectively). Phylogenetically, the group-I and group-II Mon-AstVs formed two distinct clusters, near the cluster of reptilian/amphibian AstVs, and were distantly related to avastroviruses and mamastroviruses. Since the mongooses were apparently healthy during sampling, we could not establish if the Mon-AstVs infected the animal or were of dietary origin. Although we could not ascertain the true host of the Mon-AstVs, phylogenetic analysis indicated that these viruses might have originated from lower vertebrates. To our knowledge, this is the first report on the detection and molecular characterization of AstVs in mongooses, highlighting the wide host range and significant genetic diversity within the family *Astroviridae*.

## 1. Introduction

Astroviruses (AstVs, family *Astroviridae*) are single-stranded, positive-sense RNA viruses that have been reported in a wide variety of animals [1,2,3,4]. The AstV genome (~6–7.7 kb in size) contains three major open reading frames (ORFs): ORF1a encodes a protease and other nonstructural proteins; ORF1b encodes the RNA-dependent RNA polymerase (RdRp) (facilitated by a ribosomal frameshifting signal in the overlapping region between ORF1a and -b); and ORF2 encodes the viral capsid protein [1,2,3]. In addition, some AstV genomes have been found to contain a fourth ORF, designated as ORFX (encoding a viroporin) [5].

To date, AstVs have been reported in various mammal and avian species with/without clinical illness [1,2,3,4,6]. Although AstVs were speculated to infect hosts in a species-specific manner, AstV interspecies transmission events (including cross-species recombination events) have been reported in both mammals and birds [1,2,7]. Recently, AstV genomes have been detected in lower vertebrates (amphibians, reptiles, and fish) and in invertebrates [4,8,9,10,11,12]. Furthermore, AstV-like genomes have been identified in plants [13]. Interestingly, recombination events involving the AstV capsid-encoding region have been implicated in the origin of *Hepeviridae* (a virus family that includes the hepatitis E virus) [10,14]. Taken together, these observations highlight a wide host range, and the significant genetic diversity/complex evolutionary patterns of AstVs. However, currently, the International Committee on Taxonomy of Viruses (ICTV) recognizes only two genera (genus *Mamastrovirus*, consisting of nineteen species of mammalian AstVs, and genus *Avastrovirus*, consisting of three species of avian AstVs) within the family *Astroviridae* [15], which, from the taxonomy perspective, might not reflect the overall and expanding diversity of AstVs, warranting a re-evaluation/elaboration of the classification strategy.

Astroviruses have been primarily associated with gastroenteritis in mammals, especially in humans, although AstVs have also been detected in the gut of apparently healthy animals [1,2,3,16]. Recently, AstVs have been associated with neurological symptoms in humans and some other mammals [16]. In avian species, AstVs have been associated with enteritis, hepatic disease, renal disease, systemic disease, and immunosuppression [3,16]. Although AstVs have been associated with a broad spectrum of clinical conditions, limited information is available on the mechanisms of tissue tropism, cell attachment/entry, and pathogenesis of these viruses [2,16].

Mongooses (family *Herpestidae*) are small terrestrial mammals [17,18]. Due to their invasive and scavenging behavior, mongooses have been found to stray into the habitats of humans and other animals, posing a potential risk as carriers of viral pathogens [17,19,20]. However, to date, limited information is available on the viruses circulating in mongoose populations. Mongooses have been recognized as important enzootic carriers of the rabies virus, especially in some of the Caribbean Islands, and a potential carrier of the hepatitis E virus on the island of Okinawa, Japan [19,20]. Other viruses detected in mongoose populations include adenoviruses, *Carnivore protoparvovirus* 1, cowpox virus, circoviruses, cycloviruses, feline panleukopenia virus, gemycircularviruses, picobirnavirus, and thogoto virus [21,22,23,24,25,26,27,28]. In the present study, we report, for the first-time, the detection and molecular characterization of AstVs in mongooses (the small Indian mongoose, *Urva auropunctata*).

## 2. Materials and Methods

### 2.1. Sampling

The present study was based on archival fecal samples (stored at −80 °C) obtained from the rectum/distal colon of 53 apparently healthy small Indian mongooses. During April–July 2017, a total of 83 small Indian mongooses were trapped, euthanized, and sampled during necropsy (under sterile conditions) for a gut microbiome study on the Island of St. Kitts [29]. The mongoose trapping locations on St. Kitts have been shown in a previous publication [21]. In the present study, only 53 of the 83 fecal samples were available for analysis. This study was approved by the Institutional Animal Care and Use Committee (IACUC) at the Ross University School of Veterinary Medicine, St. Kitts and Nevis (IACUC protocol numbers: 17.04.13, dated 13 April 2017, and TSU05.15.2024, dated 15 May 2024).

### 2.2. Amplification of Viral Genome

Viral RNA was extracted from the sample using the QIAamp Viral RNA Mini Kit (Qiagen Sciences, Germantown, MD, USA), following the instructions outlined by the manufacturer. The samples were screened for the presence of AstV RNA using a pan-AstV RT-hemi-nested PCR assay (targeting a short stretch (~420 bp) of the ORF1b/RdRp coding region) as described previously [30]. Additional RT-semi-nested PCRs (overlapping with the screening RT-PCR assay) were designed to obtain longer (~950 nucleotide (nt) residues) RdRp coding sequences (CDS) from the AstVs detected in the mongooses (designated as Mon-AstVs) (Supplementary Table S1 and Figure S1). The RT-PCRs were performed using the QIAGEN OneStep RT-PCR Kit (Qiagen Sciences, Germantown, MD, USA) according to the manufacturer’s instructions. To avoid contamination, sterile water was used as the negative control in all RT-PCR reactions.

### 2.3. Nucleotide Sequencing

The PCR products were purified using the Wizard^®^ SV Gel and PCR Clean-Up kit (Promega, Madison, WI, USA), following the instructions mentioned by the manufacturer. Nucleotide sequences were determined by the Sanger dideoxy method using the ABI Prism Big Dye Terminator Cycle Sequencing Ready Reaction Kit (Applied Biosystems, Foster City, CA, USA) on an ABI 3730XL Genetic Analyzer (Applied Biosystems, Foster City, CA, USA). Nucleotide sequences were determined in both directions.

### 2.4. Sequence Analysis

The AstV putative RdRp CDS and corresponding deduced amino acid (aa) sequences were determined using the ORF finder (https://www.ncbi.nlm.nih.gov/orffinder/, accessed on 11 June 2024), and confirmed by the BLASTN/BLASTX and BLASTP program (Basic Local Alignment Search Tool, www.ncbi.nlm.nih.gov/blast, accessed on 15 June 2024), respectively. Homology search for related AstV sequences was carried out using the standard BLASTN, BLASTX, and BLASTP program. Pairwise sequence identities (%) were calculated using BLASTN/BLASTP (using the “align two or more sequences option”), or the EMBOSS Needle (https://www.ebi.ac.uk/jdispatcher/psa/emboss_needle, accessed on 12 June 2024) program. Multiple alignment of the AstV RdRp aa sequences were performed using the ClustalW program (https://www.genome.jp/tools-bin/clustalw, accessed on 12 June 2024), with default parameters.

Phylogenetic analysis was carried out by the maximum likelihood (ML) method using the MEGA11 software version 11.0.13. The best-fit model of substitution (LG + G for deduced aa sequences and Tamura 3-parameter for nt sequences) was estimated using the “Find Best DNA/Protein Models (ML)” option in MEGA11. The ML trees were supported by 1000 bootstrap replicates. To rule out biases, the clustering patterns of the AstVs in ML trees were confirmed with other models of substitutions (WAG + G + I and JTT + G + F for deduced aa sequences and HKY and Kimura 2-parameter for nt sequences) as well as by the neighbor joining (NJ) method.

### 2.5. GenBank Accession Numbers

The GenBank accession numbers for the Mon-AstVs are PP977462-PP977475.

## 3. Results

The Island of St. Kitts (located in the Caribbean Lesser Antilles) is home to a large population of free-roaming small Indian mongooses that often come in close contact with humans, domesticated animals (cats, dogs, and livestock), and other wildlife species (African green monkeys, reptiles, and wild birds) [31]. In previous studies, we identified novel adenoviruses, circoviruses, cycloviruses, and picobirnaviruses in the small Indian mongoose population on St. Kitts, indicating that the island mongoose population might be harboring diverse viruses [21,22,23]. Considering this, the wide host-range of AstVs [1,2,3,4], and that there are no reports on AstVs in mongooses so far, the archival fecal samples (*n* = 53) from the small Indian mongooses on St. Kitts were screened by a pan-AstV RT-hemi-nested PCR assay (that has been successfully employed to detect genetically divergent AstVs in various animals, including wildlife species [30,32,33,34]). Fifteen (28.3%) of the 53 samples tested PCR positive for AstVs, which was confirmed by sequencing of the PCR amplicons. 

**Figure 1:**
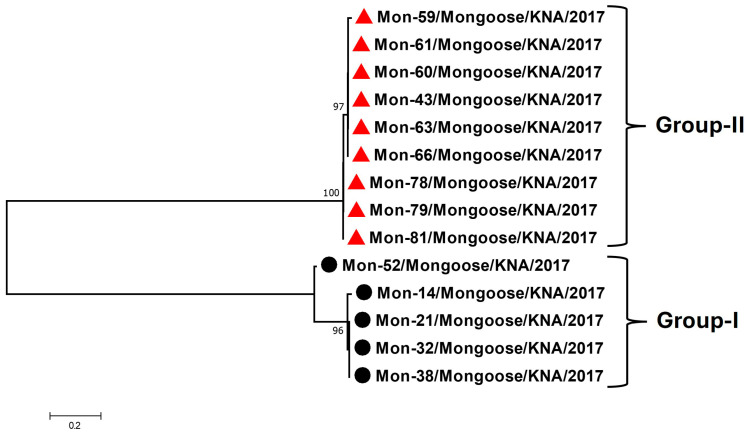
Phylogenetic analysis of the partial RdRp CDS (~400 nt, obtained from the screening RT-PCR) of AstVs in mongooses (designated as Mon-AstVs). The group-I and group-II Mon-AstVs are shown with black circles and red triangles, respectively. The name of the virus/host of detection/year of sampling have been mentioned for the Mon-AstVs. Bootstrap values < 70% are not shown. Scale bar, 0.2 substitutions per nt. Abbreviations: AstV, astrovirus; CDS, coding sequence; nt, nucleotide; RdRp, RNA-dependent RNA polymerase.

Based on nt sequence identities and phylogenetic analysis of the partial RdRp CDS (~400 nt, obtained from the screening RT-PCR), the Mon-AstVs were classified into two distinct groups: group-I (consisting of Mon-14, -21, -32, -38, and -52) and group-II (Mon-43, -59, -60, -61, -63, -66, -78, -79, and -81) (Figure 1). The RdRp sequence obtained from a single AstV positive sample (sample Mon-9) lacked quality (phred value < 40) and was not included in the analysis. The group-I and group-II Mon-AstVs shared nt sequence identities of 92–100% and 98–100%, respectively, within a group, while identities of ~63–64% were observed between the two groups. With other AstVs, the group-I and group-II Mon-AstV sequences shared maximum (yet low) nt identities (66.4–68.5% and ~65%, respectively) with cognate sequences of AstVs from reptilian (Hainan gekko similignum AstV (GenBank accession number MG599917) and Hetplan gecko AstVs (PP711188 and PP711198)) and amphibian (Zhejiang Chinese fire belly newt AstV (MG599916)) species.

**Figure 2:**
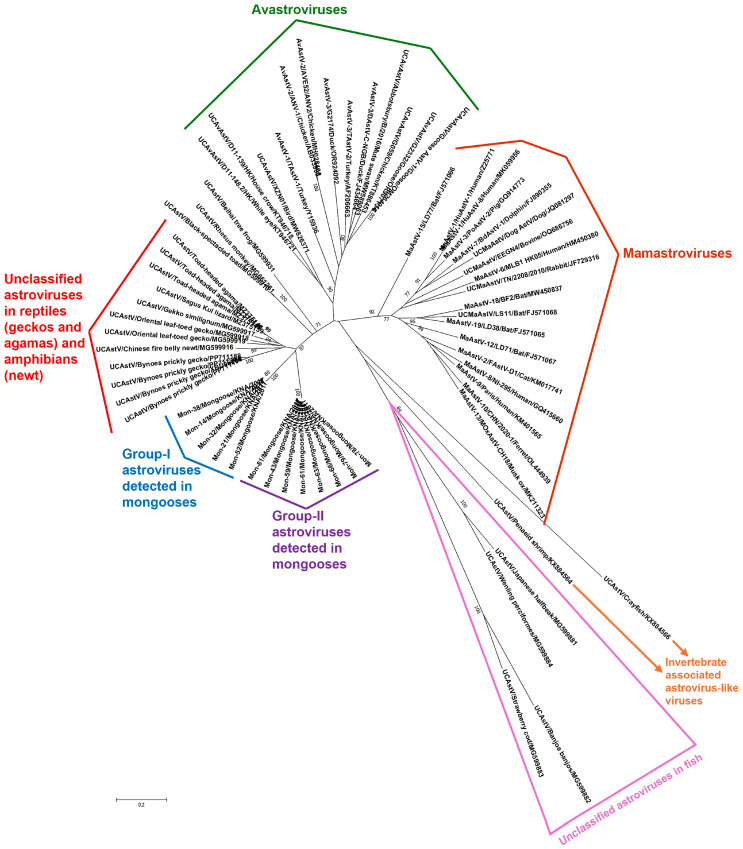
Phylogenetic analysis of the partial deduced aa sequences (~315 aa) of putative RdRps of the group-I and group-II AstVs detected in mongooses (designated as Mon-AstVs) with those of AstVs from other host species. The ‘name of the virus/detected in animal/year of sampling’ has been mentioned for the Mon-AstVs, while the ‘name of the virus/strain, or isolate/host/GenBank accession number’ and the ‘name of the virus/detected in animal/GenBank accession number’ have been shown for the AvAstVs/MaAstVs and other AstVs (from reptiles, amphibians, fish, and invertebrates), respectively. The details of the AstVs within the different clusters are shown in Supplementary Table S2. Since some of the taxon names overlap/are compressed in the radial tree, a rectangular version (rectangular phylogenetic tree) of Figure 2 has been shown in Supplementary Figure S2. Bootstrap values < 70% are not shown. Scale bar, 0.2 substitutions per aa. Abbreviations: aa, amino acid; AstVs: astroviruses; AvAstV, avastrovirus; MaAstV, mamastrovirus; RdRp, RNA-dependent RNA polymerase; UCAstV, unclassified AstV.

To gain a better understanding of the genetic make-up/diversity of the Mon-AstVs, longer RdRp CDS (~950 bp, encoding a significant region (~315 aa) of the putative RdRp) were obtained from the Mon-AstVs (except for Mon-9 and -60, as samples were exhausted) by combining the screening RT-PCR with additional RT-semi-nested PCR assays (Supplementary Table S1 and Figure S1). The partial deduced aa sequences (~315 aa) of the putative RdRps of group-I and group-II Mon-AstVs shared identities of 97.77–100% and 99.68–100%, respectively, within a group, while deduced aa identities of 66.45–67.30% were observed between the groups. With AstVs from other host species, the group-I and group-II Mon-AstV RdRp sequences shared maximum deduced aa identities of 67.82–68.35% and 66.77–66.99%, respectively, with the cognate sequence of unclassified AstV isolate 5PT-RDRP-5 (GenBank accession number MZ375145) from a toad-headed agama (*Phrynocephalus theobaldi*), followed by identities of 61.29–68.04% and 63.14–66.67%, respectively, with other unclassified AstVs from reptilian (agamas and geckos) and amphibian (newt) species. The putative RdRps of the Mon-AstVs shared low deduced aa identities of <54% and <50% with those of avastroviruses and mamastroviruses, respectively. By phylogenetic analysis of partial RdRp sequences, the group-I and group-II Mon-AstVs formed two distinct clusters, near the cluster of reptilian and amphibian AstVs, and were distantly related to avastroviruses and mamastroviruses (Figure 2 and Supplementary Figure S2). Although the putative RdRps of the Mon-AstVs appeared to be distinct from those of other AstVs, they retained the various features (catalytic core domain containing the polymerase motifs A, -B, and -C) that are conserved among RdRps within the family *Astroviridae* (Supplementary Figure S3) [35,36].

## 4. Discussion

To our knowledge, this is the first report on the detection and molecular characterization of AstVs in mongooses, highlighting the expanding and wide variety of animal species from which the virus has been reported [1,2,3,4]. The high AstV detection rates (28.3%, 15/53) in the small Indian mongoose population on St. Kitts Island indicated that AstVs might be widely circulating in mongoose populations, warranting similar studies in other mongoose species and in other geographical regions. The identification of the two genetically divergent Mon-AstV groups (group-I and group-II) in a small (~69 square miles), isolated geographical region revealed the significant genetic diversity among AstVs in the St. Kitts small Indian mongoose population (Figure 1 and Figure 2). Based on sequence identities and phylogenetic analysis of the partial RdRp sequences, we could not ascertain the true origin of the AstVs from the mongooses, as they appeared to be distinct from AstVs reported in other host species (Figure 2 and Supplementary Figure S2). However, phylogenetically, the putative RdRps of the Mon-AstVs appeared to be more related to those of unclassified AstVs from reptiles and amphibian species than those of other AstVs, indicating that the Mon-AstVs might share a common ancestry with reptilian/amphibian AstVs (Figure 2 and Supplementary Figure S2). 

Although AstVs are considered host-specific, evidence in support of inter-species transmission events, including those between distantly related animal taxa, has been reported in higher vertebrates [7]. In a study from China, AstV sequences obtained from a simian fecal sample were found to be closely related to those of amphibian-associated AstVs, indicating possible cross-species transmission events involving amphibian and mammalian species, although it is more likely that dietary/environmental contamination resulted in these findings [37]. In the present study, phylogenetic analysis (supported by deduced aa identities) suggested that the AstVs detected in the mongooses might have originated from lower vertebrates (Figure 2 and Supplementary Figure S2). Environmental contamination of the mongoose fecal samples was unlikely, as the small Indian mongooses were sampled during necropsy under sterile conditions. Considering the feeding habits of the small Indian mongoose (a polyphagous predator that feeds on reptiles and amphibians [38]), it might be possible that the Mon-AstVs were of dietary origin. Since the mongooses were apparently healthy during sampling and animal tissue samples were not screened for AstVs, we could not determine if the Mon-AstVs infected the host.

Frequent host-switching events on the background of long-term virus–host co-divergence have been proposed to play important roles in the evolution of vertebrate RNA viruses, and such events are influenced by host ecology and behavior [9,10,39,40]. Interestingly, in this regard, the diverse RNA virosphere in lower vertebrates was found to consist of certain RNA viruses that are related to known viral pathogens of higher vertebrates, and some of these viruses exhibited similar tissue tropisms as their mammalian counterparts [9,10,39]. Moreover, it has been hypothesized that interactions between AstVs and the gut microbiome might have detrimental implications for the host [41,42]. Considering these observations, the detection of Mon-AstVs (possibly derived from lower vertebrates) in the gut of a mammalian species warrants further studies on the evolution/origin, tissue tropism, and pathogenesis of these viruses. 

Although the present study is the first to report genetically divergent AstVs in the mongoose, there were limitations: (i) although we reported high AstV detection rates (28.3%), the study was based on a small sample size (*n* = 53), (ii) since the mongoose samples have been used in previous studies [21,22,23], they were not available in sufficient volumes for further analysis, such as next generation sequencing, which would have provided more information on the genetic makeup of these AstVs, and (iii) we could not amplify other regions of the Mon-AstV ORF1ab (than those reported here) using degenerate primers that were designed following multiple alignment of published AstV ORF1ab sequences (including those from reptiles and amphibians), while the reptilian/amphibian AstV ORF2 sequences were too divergent to design primers for RT-PCR assays targeting the capsid encoding region. 

## Data Availability

The data presented in this study are available in this article, Supplementary Tables S1 and S2, and Supplementary Figures S1–S3.

## Supplementary Materials

The following supporting information can be downloaded at: https://www.mdpi.com/article/10.3390/v16081269/s1, Figure S1: A schematic representation of the overlapping RT-PCRs used to obtain longer ORF1b sequences (~950 bp, encoding a significant region (~315 aa) of the putative RdRp) from the AstVs detected in mongooses (designated as Mon-AstVs); Figure S2: Rectangular version (rectangular phylogenetic tree) of the radial phylogenetic tree shown in Figure 2; Figure S3: Multiple alignment of the partial deduced aa sequences (~315 aa) of putative RdRps of the AstVs detected in mongooses (designated as Mon-AstVs) with those of other AstVs; Table S1: Additional RT-semi-nested PCRs (overlapping with the screening RT-PCR assay) used in combination with the screening RT-PCR to obtain longer ORF1b sequences (~950 bp, encoding a significant region (~315 aa) of the putative RdRp) from the AstVs detected in mongooses (designated as Mon-AstVs); Table S2: Astrovirus (AstV) strains within the different clusters in Figure 2.
