# Supplementary material for: First Report on Detection and Molecular Characterization of Astroviruses in Mongooses
Source: Viruses. 2024 Aug 8;16(8):1269. doi: 10.3390/v16081269 (PMC11358933; doi:10.3390/v16081269)

Supplementary Figure S1. A schematic representation of the overlapping RT-PCRs used to obtain longer ORF1b sequences (~950 bp, encoding a significant region (~ 315 aa) of the putative RdRp) from the AstVs detected in mongooses (designated as Mon-AstVs). The nt positions shown here are those at corresponding positions of the nt sequence of Hainan gekko similignum astrovirus strain LPXYC188631 (GenBank accession number MG599917). Abbreviations: aa, amino acid; AstVs, astroviruses; nt, nucleotide; ORF, open reading frame; RdRp, RNA-dependent RNA polymerase.

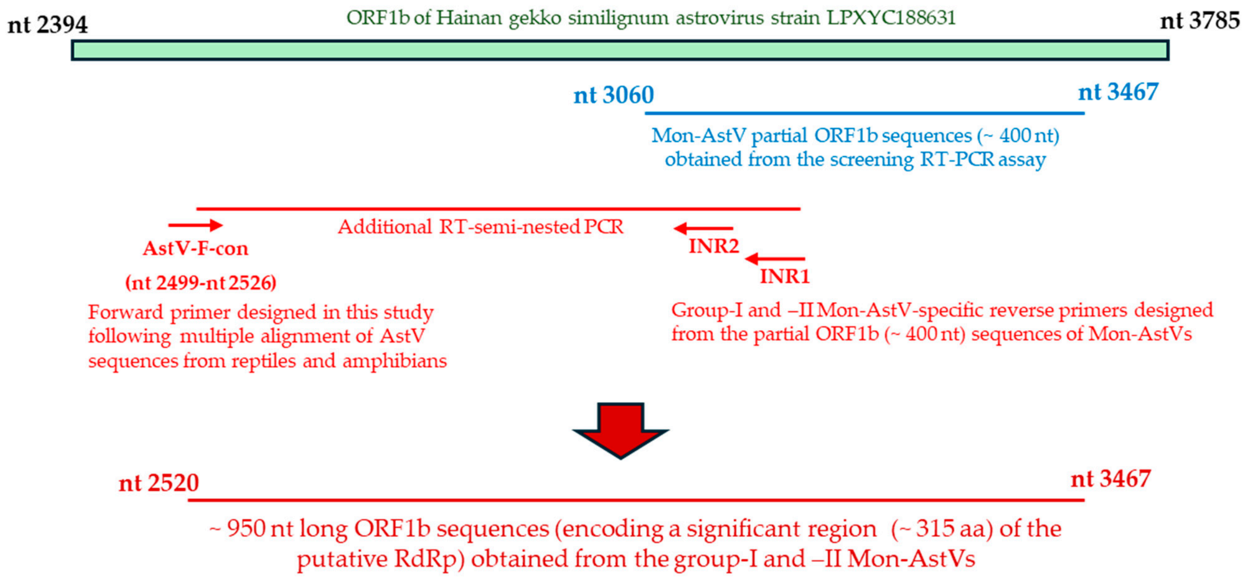

Supplement: Supplementary file 1 [file viruses-16-01269-s001.zip › Supplementary Figure S1.pdf]
