# Supplementary material for: First Report on Detection and Molecular Characterization of Astroviruses in Mongooses
Source: Viruses. 2024 Aug 8;16(8):1269. doi: 10.3390/v16081269 (PMC11358933; doi:10.3390/v16081269)

Supplementary Figure S2. Rectangular version (rectangular phylogenetic tree) of the radial phylogenetic tree shown in figure 2. Phylogenetic analysis of the partial deduced aa sequences (~ 315 aa) of the putative RdRps of group-I and -II AstVs detected in mongooses (designated as Mon-AstVs) with those of AstVs from other host species. The 'name of the virus/detected in animal/year of sampling' have been mentioned for the Mon-AstVs, whilst the 'name of the virus/strain, or isolate/host/GenBank accession number' and the 'name of the virus/detected in animal/GenBank accession number' have been shown for the AvAstVs/MaAstVs and other AstVs (from reptiles, amphibians, fish, and invertebrates), respectively. Bootstrap values < 70% are not shown. Scale bar, 0.2 substitutions per aa. Abbreviations: aa, amino acid; AstVs: astroviruses; AvAstV, avastrovirus; MaAstV, mamastrovirus; RdRp, RNA-dependent RNA polymerase; UCAstV, unclassified AstV.

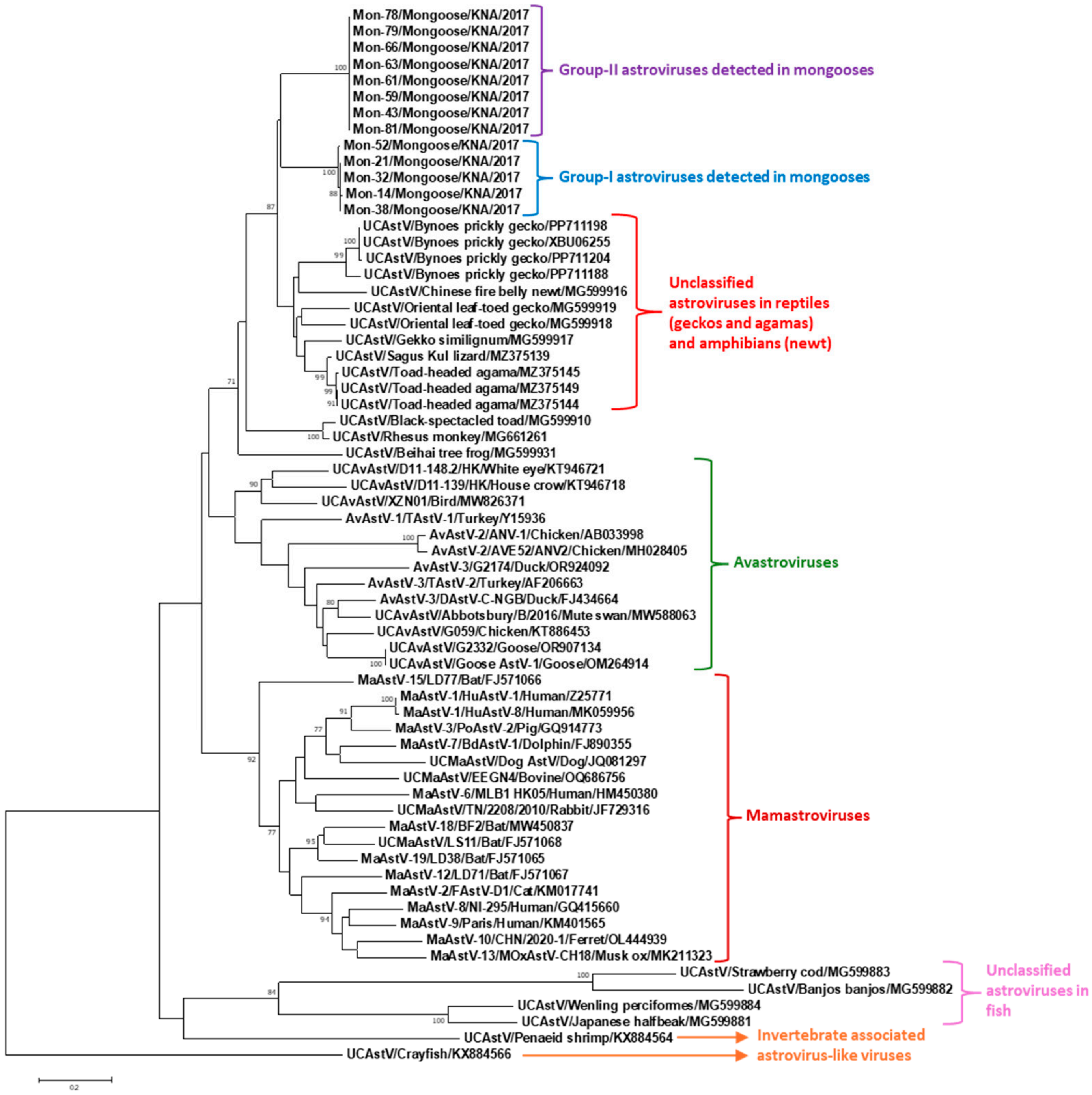

Supplement: Supplementary file 1 [file viruses-16-01269-s001.zip › Supplementary Figure S2.pdf]
