# Supplementary material for: First Report on Detection and Molecular Characterization of Astroviruses in Mongooses
Source: Viruses. 2024 Aug 8;16(8):1269. doi: 10.3390/v16081269 (PMC11358933; doi:10.3390/v16081269)
Supplement: Supplementary file 1 [file viruses-16-01269-s001.zip › Supplementary Figure S3.pdf]

Supplementary Figure S3. Multiple alignment of the partial deduced aa sequences (~ 315 aa) of putative RdRps of the AstVs detected in mongooses (designated as Mon-AstVs) with those of other AstVs. The Mon-AstVs retained the putative RdRp catalytic core domain (shown with underline) and the polymerase motif A, -B and -C (highlighted with yellow, blue, and green, respectively) that are conserved among RdRps within the family *Astroviridae*. The conserved catalytic residues ('D' within polymerase motif A, and 'GDD' within polymerase motif C) are shown with the '#" symbol. A '-' indicates absence of an 'aa' residue at that position. The 'group/name of the virus/detected in animal' have been shown for the Mon-AstVs, whilst the 'name of the virus/host/GenBank accession number' have been mentioned for the other AstVs. Positions of aa residues correspond to those of the putative ORF1ab product of MaAstV-8/Human/GQ415660. Abbreviations: aa, amino acid; AstV, astrovirus; AvAstV, avastrovirus; MaAstV, mamastrovirus; RdRp, RNA-dependent RNA polymerase; UCAstV, unclassified AstV.

```
                                960
MaAstV-8/Human/GQ415660          KFTYKEPMSNIKDKYKREWNFAMRVLRREFDYLVDSVMTDITATSKNSDSTPSYPKCLWW
AvAstV-2/Chicken/AB033998        KFDYGDAVD-FVQDYPELTAFADAAVLAEVGYMEGTHVIPIQETSKNMDSTPAFPKMLDF
UCAstV/Agama/MZ375145            KFTYREPCEDIKNKYREEWDFATYCVLMEYSYMQDTKLTPIWYTTKNEDSIPGYPKFLYW
UCAstV/Gecko/MG599919            KFVYREPLDDIKNKYRPEWDFATYCVLIEFSYMTDSRLVPIGSTSKNQDSIPGYPKFFWF
Group-I/Mon-14/Mongoose          KFIYREPMDNIAQKFPLEWAFATEVVLREYSYMQDTDLLDMAYTTKNPNSTPGYPKFYYW
Group-I/Mon-21/Mongoose          KFIYREPMDNIAQKFPLEWAFATEVVLREYSYMQDTDLLDMAYTTKNPNSTPGYPKFYYW
Group-I/Mon-32/Mongoose          KFIYREPMDNIAQKFPLEWAFATEVVLREYSYMQDTDLLDMAYTTKNPNSTPGYPKFYYW
Group-I/Mon-38/Mongoose          KFIYREPMDNIAQKFPLEWAFATEVVLREYSYMQDTDLLDMAYTTKNPNSTPGYPKFYYW
Group-I/Mon-52/Mongoose          KFIYREPMDNIAQKFPLEWAFATEVVLREYSYMQDTDLLDMAYTTKNPNSTPGYPKFYYW
Group-II/Mon-43/Mongoose         -FIYREPKHGIRHLYPEEWDFATEVVLTEYSYMQDTHIIPISSTTKNVDSTPGYPKFEYW
Group-II/Mon-59/Mongoose         KFIYREPKHGIRHLYPEEWDFATEVVLTEYSYMQDTHIIPISSTTKNVDSTPGYPKFEYW
Group-II/Mon-61/Mongoose         --IYREPKHGIRHLYPEEWDFATEVVLTEYSYMQDTHIIPISSTTKNVDSTPGYPKFEYW
Group-II/Mon-63/Mongoose         -FIYREPKHGIRHLYPEEWDFATEVVLTEYSYMQDTHIIPISSTTKNVDSTPGYPKFEYW
Group-II/Mon-66/Mongoose         KFIYREPKHAIRHLYPEEWDFATEVVLTEYSYMQDTHIIPISSTTKNVDSTPGYPKFEYW
Group-II/Mon-78/Mongoose         -FIYREPKHGIRHLYPEEWDFATEVVLTEYSYMQDTHIIPISSTTKNVDSTPGYPKFEYW
Group-II/Mon-79/Mongoose         KFIYREPKHGIRHLYPEEWDFATEVVLTEYSYMQDTHIIPISSTTKNVDSTPGYPKFEYW
Group-II/Mon-81/Mongoose         KFIYREPKHGIRHLYPEEWDFATEVVLTEYSYMQDTHIIPISSTTKNVDSTPGYPKFEYW


MaAstV-8/Human/GQ415660          KTEAEYLKDRGYQDYITQLESIKKGE-RPRVLWYLFLKKEILKLSKIEDGDIRQIVCSDP
AvAstV-2/Chicken/AB033998        DSERDYLEAHGMKEYIDTQLGVQ----SGKPLWWCFLKNEILKEKKVSEDDIRIITCSDP
UCAstV/Agama/MZ375145            QTERDYIQDCGRSEYMSVWQQISDKTFDLSPVWYSFLKLEPTTFEKIEAEDYRMIMCTDP
UCAstV/Gecko/MG599919            STEKEYLDKCGQREYFQLWDKIKDPEFTESPVWYSFLKMEPTTLDKIESEDYRMIMCTDP
Group-I/Mon-14/Mongoose          NDEEEYVRDCGMDEYYEVWRQISKPC-KKVPVWWSFLKLEPTLEEKIREENYRMIMCTDP
Group-I/Mon-21/Mongoose          NDEEEYVRDCGMDEYYEVWRQISKPC-KKVPVWWSFLKLEPTLEEKIREENYRMIMCTDP
Group-I/Mon-32/Mongoose          NDEEEYVRDCGMDEYYEVWRQISKPC-KKVPVWWSFLKLEPTLEEKIREENYRMIMCTDP
Group-I/Mon-38/Mongoose          NDEEEYVRDCGMDEYYEVWRQISKPC-KKVPVWWSFLKLEPTLEEKIREENYRMIMCTDP
Group-I/Mon-52/Mongoose          NDEEEYVRDCGMDEYYEVWRQISKPC-KKVPVWWSFLKLEPTLEEKIREENYRMIMCTDP
Group-II/Mon-43/Mongoose         DSEYSYLEECGFREYYETWDQIKDKDFDENPLWWSFLKMETTKQEKIKQQDYRMIMCTDP
Group-II/Mon-59/Mongoose         DSEYSYLEECGFREYYETWDQIKDKDFDENPLWWSFLKMETTKQEKIKQQDYRMIMCTDP
Group-II/Mon-61/Mongoose         DSEYSYLEECGFREYYETWDQIKDKDFDENPLWWSFLKMETTKQEKIKQQDYRMIMCTDP
Group-II/Mon-63/Mongoose         DSEYSYLEECGFREYYETWDQIKDKDFDENPLWWSFLKMETTKQEKIKQQDYRMIMCTDP
Group-II/Mon-66/Mongoose         DSEYSYLEECGFREYYETWDQIKDKDFDENPLWWSFLKMETTKQEKIKQQDYRMIMCTDP
Group-II/Mon-78/Mongoose         DSEYSYLEECGFREYYETWDQIKDKDFDENPLWWSFLKMETTKQEKIKQQDYRMIMCTDP
Group-II/Mon-79/Mongoose         DSEYSYLEECGFREYYETWDQIKDKDFDENPLWWSFLKMETTKQEKIKQQDYRMIMCTDP
Group-II/Mon-81/Mongoose         DSEYSYLEECGFREYYETWDQIKDKDFDENPLWWSFLKMETTKQEKIKQQDYRMIMCTDP

                                                                                        #
MaAstV-8/Human/GQ415660          IFARIGCVFEEHQNNLMKMRTKTRMGQCGWSPFCGGFNDRVKRLVDKGNNLFVEFDWTRY
AvAstV-2/Chicken/AB033998        VITRLGASFDSEQNERMKERTETHHAQVGWTPFFGGLDKRVRRITSCGRTQVLELDWTRF
UCAstV/Agama/MZ375145            VYTRVGSVFEQHQNELMKQHTYQRAGQMGWTPFKGGLDMTLRR-ISEGAEYIMELDWTRF
UCAstV/Gecko/MG599919            CYTRIGAVFEQHQNELMKEHTHQRAGQMGWCPFKGGLDRTLRR-ISSGMTKFLELDWTRF
Group-I/Mon-14/Mongoose          VFTRIGACFEQHQNELMKAHTRTHAGQVGWTPFKGGLDETLRR-IAKDSECFLELDWTRF
Group-I/Mon-21/Mongoose          VFTRIGACFEQHQNELMKAHTRTHAGQVGWTPFKGGLDETLRR-IAKDSECFLELDWTRF
Group-I/Mon-32/Mongoose          VFTRIGACFEQHQNELMKAHTRTHAGQVGWTPFKGGLDETLRR-IAKDSECFLELDWTRF
Group-I/Mon-38/Mongoose          VFTRIGACFEQHQNELMKAHTRTHAGQVGWTPFKGGLDETLRR-IAKDSECFLELDWTRF
Group-I/Mon-52/Mongoose          VFTRIGACFEQHQNELMKAHTRTHAGQVGWTPFKGGLDETLRR-IAKDSECFLELDWTRF
Group-II/Mon-43/Mongoose         VFTRIGACFEQHQNELMKSHTLQKAGQMGWTPFKGGIDYTLRR-IAKNSQYFLELDWTRF
Group-II/Mon-59/Mongoose         VFTRIGACFEQHQNELMKSHTLQKAGQMGWTPFKGGIDYTLRR-IAKNSQYFLELDWTRF
Group-II/Mon-61/Mongoose         VFTRIGACFEQHQNELMKSHTLQKAGQMGWTPFKGGIDYTLRR-IAKNSQYFLELDWTRF
Group-II/Mon-63/Mongoose         VFTRIGACFEQHQNELMKSHTLQKAGQMGWTPFKGGIDYTLRR-IAKNSQYFLELDWTRF
Group-II/Mon-66/Mongoose         VFTRIGACFEQHQNELMKSHTLQKAGQMGWTPFKGGIDYTLRR-IAKNSQYFLELDWTRF
Group-II/Mon-78/Mongoose         VFTRIGACFEQHQNELMKSHTLQKAGQMGWTPFKGGIDYTLRR-IAKNSQYFLELDWTRF
Group-II/Mon-79/Mongoose         VFTRIGACFEQHQNELMKSHTLQKAGQMGWTPFKGGIDYTLRR-IAKNSQYFLELDWTRF
Group-II/Mon-81/Mongoose         VFTRIGACFEQHQNELMKSHTLQKAGQMGWTPFKGGIDYTLRR-IAKNSQYFLELDWTRF
```

```
MaAstV-8/Human/GQ415660         DGTIPNEVFMAIKQFRYSCLAEEFKTEENLSIYKWYCESILDRYVMLPSGEVTKQVRGNP
AvAstV-2/Chicken/AB033998       DGTIPVQLFQRMRELRKFFLTRRSRRR-YGKLLDWYNAQLTDRITLLPTGEVTHVKKGNP
UCAstV/Agama/MZ375145           DGTIPQEVLRHIKSIRWFFLDGEYKTTENYIRHQWYVDQLCNKYVVLPTGEVTLIGRGNP
UCAstV/Gecko/MG599919           DGTIPAEVLLHIKNIRWFLLDPAYKTPENWQRHKWYVEQLVNKYVLIPTGEVTMIDRGNP
Group-I/Mon-14/Mongoose         DGTIPAEVLRHIKEIRYFFLDRKYHTDENRRRHEWYVDSILNKFVVLPTGEVTIIRRGNP
Group-I/Mon-21/Mongoose         DGTIPAEVLRHIKEIRYFFLDRKYHTDEDRRRHEWYVDSILNKFVVLPTGEVTIIRRGNP
Group-I/Mon-32/Mongoose         DGTIPAEVLRHIKEIRYFFLDRKYHTDENRRRHEWYVDSILNKFVVLPTGEVTIIRRGNP
Group-I/Mon-38/Mongoose         DGTIPAEVLRHIKEIRYFFLDRKYHTDENRRRHEWYVDSILNKFVVLPTGEVTIIRRGNP
Group-I/Mon-52/Mongoose         DGTIPAEVLRHIKEIRYFFLDRKYHTDENRRRHEWYVDSILNKFVVLPTGEVTIIRRGNP
Group-II/Mon-43/Mongoose        DGTIPSEVYFHIKQLRFFFLHEVHHTRENWNRYQWYVKNLVQKYVVIPTGEVTMISRGNP
Group-II/Mon-59/Mongoose        DGTIPSEVYFHIKQLRFFFLHEVHHTRENWNRYQWYVKNLVQKYVVIPTGEVTMISRGNP
Group-II/Mon-61/Mongoose        DGTIPSEVYFHIKQLRFFFLHEVHHTRENWNRYQWYVKNLVQKYVVIPTGEVTMISRGNP
Group-II/Mon-63/Mongoose        DGTIPSEVYFHIKQLRFFFLHEVHHTRENWNRYQWYVKNLVQKYVVIPTGEVTMISRGNP
Group-II/Mon-66/Mongoose        DGTIPSEVYFHIKQLRFFFLHEVHHTRENWNRYQWYVKNLVQKYVVIPTGEVTMISRGNP
Group-II/Mon-78/Mongoose        DGTIPSEVYFHIKQLRFFFLHEVHHTRENWNRYQWYVKNLVQKYVVIPTGEVTMISRGNP
Group-II/Mon-79/Mongoose        DGTIPSEVYFHIKQLRFFFLHEVHHTRENWNRYQWYVKNLVQKYVVIPTGEVTMISRGNP
Group-II/Mon-81/Mongoose        DGTIPSEVYFHIKQLRFFFLHEVHHTRENWNRYQWYVKNLVQKYVVIPTGEVTMISRGNP

                                                                                ###
MaAstV-8/Human/GQ415660         SGQVSTTMDNNLCNVFFQAFEYAYMHPD-KDISQLMHD-WEKVDSLIYGDDRLSTY-PEL
AvAstV-2/Chicken/AB033998       SGQFSTTVDNNLVNEWLTAFEFGYQHLENHGIIPTVRDYRANVDFLCYGDDRLLAFNPSF
UCAstV/Agama/MZ375145           SGQISTTSDNIMVNTFLTAFEYAYQAFR-TGRDPVPEDFFRDHKMICYGDDRLSGI-VGY
UCAstV/Gecko/MG599919           SGQISTTSDNIFVNVFLTAFEYVYQAER-EGRTVTPEQFFMDHKMVCYGDDRLSGV--NY
Group-I/Mon-14/Mongoose         SGQISTTTDNIMVNTFLTAFEYIYQNKF-QGRRLTPDMFFKEHKLICYGDDRLSGI-NGY
Group-I/Mon-21/Mongoose         SGQISTTTDNIMVNTFLTAFEYIYQNKF-QGRRVTPDMFFKEHKMICYGDDRLSGI-NGY
Group-I/Mon-32/Mongoose         SGQISTTTDNIMVNTFLTAFEYIYQNKF-QGRRVTPDMFFKEHKMICYGDDRLSGI-NGY
Group-I/Mon-38/Mongoose         SGQISTTTDNIMVNTFLTAFEYIYQNKF-QGRRVTPDMFFKEHKMICYGDDRLSGI-NGY
Group-I/Mon-52/Mongoose         SGQISTTTDNIMVNTFLTAFEYIYQNKF-QGRRVTPDMFFREHKMICYGDDRLSGI-SGY
Group-II/Mon-43/Mongoose        SGQISTTSDNIMVNTFLTAFEYKYQALR-EGRVVKIQDFFKEHKSICYGDDRLSGV--SY
Group-II/Mon-59/Mongoose        SGQISTTSDNIMVNTFLTAFEYKYQALR-EGRVVKIQDFFKEHKSICYGDDRLSGV--SY
Group-II/Mon-61/Mongoose        SGQISTTSDNIMVNTFLTAFEYKYQALR-EGRVVKIQDFFKEHKSICYGDDRLSGV--SY
Group-II/Mon-63/Mongoose        SGQISTTSDNIMVNTFLTAFEYKYQALR-EGRVVKIQDFFKEHKSICYGDDRLSGV--SY
Group-II/Mon-66/Mongoose        SGQISTTSDNIMVNTFLTAFEYKYQALR-EGRVVKIQDFFKEHKSICYGDDRLSGV--SY
Group-II/Mon-78/Mongoose        SGQISTTSDNIMVNTFLTAFEYKYQALR-EGRVVKIQDFFKEHKSICYGDDRLSGV--SY
Group-II/Mon-79/Mongoose        SGQISTTSDNIMVNTFLTAFEYKYQALR-EGRVVKIQDFFKEHKSICYGDDRLSGV--SY
Group-II/Mon-81/Mongoose        SGQISTTSDNIMVNTFLTAFEYKYQALR-EGRVVKIQDFFKEHKSICYGDDRLSGV--SY

                                                  1274
MaAstV-8/Human/GQ415660         PEDYVDRVVDMYATVFGMW
AvAstV-2/Chicken/AB033998       VNYDPQVTIDMYKNIFGMW
UCAstV/Agama/MZ375145           VP-DVDMIVDMYEKVFGMW
UCAstV/Gecko/MG599919           EP-NIDMIVKMYADIFGMW
Group-I/Mon-14/Mongoose         VP-DRNLLISMYENIYGMW
Group-I/Mon-21/Mongoose         VP-DRNLLISMYENIFGMW
Group-I/Mon-32/Mongoose         VP-DRNLLISMYENIFGMW
Group-I/Mon-38/Mongoose         VP-DRNLLISMYENIFGM-
Group-I/Mon-52/Mongoose         TP-DRNLLISMYESIFGMW
Group-II/Mon-43/Mongoose        LP-NKELIIDMYEQIFGM-
Group-II/Mon-59/Mongoose        LP-NKELIIDMYEQIFGMW
Group-II/Mon-61/Mongoose        LP-NKELIIDMYEQIFGMW
Group-II/Mon-63/Mongoose        LP-NKELIIDMYEQIFGMW
Group-II/Mon-66/Mongoose        LP-NKELIIDMYEQIFGMW
Group-II/Mon-78/Mongoose        LP-NKELIIDMYEQIFGM-
Group-II/Mon-79/Mongoose        LP-NKELIIDMYEQIFGMW
Group-II/Mon-81/Mongoose        LP-NKELIIDMYEQIFGMW
```
