# Supplementary material for: First Report on Detection and Molecular Characterization of Astroviruses in Mongooses
Source: Viruses. 2024 Aug 8;16(8):1269. doi: 10.3390/v16081269 (PMC11358933; doi:10.3390/v16081269)
Supplement: Supplementary file 1 [file viruses-16-01269-s001.zip › Supplementary Table S1.pdf]

Supplementary Table S1. Additional RT-semi-nested PCRs (overlapping with the screening RT-PCR assay) used in combination with the screening RT-PCR to obtain longer ORF1b sequences (~950 bp, encoding a significant region (~ 315 aa) of the putative RdRp) from the AstVs detected in mongooses (designated as Mon-AstVs). Abbreviations: aa, amino acid; AstVs, astroviruses; nt, nucleotide; ORF, open reading frame; RdRp, RNA-dependent RNA polymerase.

**Target**	**RT-semi-nested PCR**	**Primer name**	**Primer sequence (5′-3′)**	**Primer position**
ORF1b (RdRp encoding region)	Group-I Mon-AstV-specific RT-semi-nested PCR	AstV-F-con	GCATATDMWAAKATGTTTGARAAATTTA [1]	nt 2499-nt 2526 [2]
		Group-I-INR1	GGTAGTACTAATCTGACCAGAAGG [3]	nt 199-nt 176 [4]
		Group-I-INR2	CTCCTTATTATGGTTACTTCCC [3]	nt 168-nt 147 [4]
	Group-II Mon-AstV-specific RT-semi-nested PCR	AstV-F-con	GCATATDMWAAKATGTTTGARAAATTTA [1]	nt 2499-nt 2526 [2]
		Group-II-INR1	GTAGTTGAAATTTGTCCCGAGGG [5]	nt 195-nt 173 [6]
		Group-II-INR2	ATCATGGTAACCTCTCCTGTAGG [5]	nt 159-nt 137 [6]

[1] The primer was designed in the present study following multiple alignment of AstV sequences from reptiles and amphibians.

[2] The primer positions are those of nt sequences of Hainan gekko similignum astrovirus strain LPXYC188631 (GenBank accession number MG599917)

[3] The primer was designed in the present study following multiple alignment of AstV sequences from group-I Mon-AstVs.

[4] The primer positions are those of the partial nt sequence (~ 400 nt, obtained from the screening PCR) of group-I Mon-AstV Mon-14.

[5] The primer was designed in the present study following multiple alignment of AstV sequences from group-II Mon-AstVs.

[6] The primer positions are those of the partial nt sequence (~ 400 nt, obtained from the screening PCR) of group-II Mon-AstV Mon-59.
