# Supplementary material for: First Report on Detection and Molecular Characterization of Astroviruses in Mongooses
Source: Viruses. 2024 Aug 8;16(8):1269. doi: 10.3390/v16081269 (PMC11358933; doi:10.3390/v16081269)
Supplement: Supplementary file 1 [file viruses-16-01269-s001.zip › Supplementary Table S2.pdf]

Supplementary Table S2. Astrovirus (AstV) strains within the different clusters in figure 2.

**Cluster of group-I AstVs detected in mongooses**
Mon-14/Mongoose/KNA/2017 (name of virus/detected in animal/country/year of sampling)
Mon-21/Mongoose/KNA/2017
Mon-32/Mongoose/KNA/2017
Mon-38/Mongoose/KNA/2017
Mon-52/Mongoose/KNA/2017
**Cluster of group-II AstVs detected in mongooses**
Mon-43/Mongoose/KNA/2017 (name of virus/detected in animal/country/year of sampling)
Mon-59/Mongoose/KNA/2017
Mon-61/Mongoose/KNA/2017
Mon-63/Mongoose/KNA/2017
Mon-66/Mongoose/KNA/2017
Mon-78/Mongoose/KNA/2017
Mon-79/Mongoose/KNA/2017
Mon-81/Mongoose/KNA/2017
**Cluster of unclassified AstVs in reptiles and amphibians:**
UCAstV/Toad-headed agama/MZ375145 (unclassified AstV/detected in animal/GenBank accession number)
UCAstV/Toad-headed agama/MZ375149
UCAstV/Toad-headed agama/MZ375144
UCAstV/Sagus Kul lizard/MZ375139
UCAstV/Chinese fire belly newt/MG599916
UCAstV/Oriental leaf-toed gecko/MG599919
UCAstV/Oriental leaf-toed gecko/MG599918
UCAstV/Gekko similignum/MG599917
UCAstV/Bynoe's prickly gecko/PP711198
UCAstV/Bynoe's prickly gecko/PP711188
UCAstV/Bynoe's prickly gecko/PP711204
UCAstV/Bynoe's prickly gecko/XBU06255
**Astroviruses forming distinct branches near the reptilian-amphibian AstV cluster:**
UCAstV/Black-spectacled toad/MG599910 (unclassified AstV/detected in animal/GenBank accession number)
UCAstV/Rhesus monkey/MG661261
UCAstV/Beihai tree frog/MG599931
**Cluster of avastroviruses:**
AvAstV-1/TAstV-1/Turkey/Y15936 (Avastrovirus-1/strain, or isolate/host/GenBank accession number)
AvAstV-2/ANV-1/Chicken/AB033998
AvAstV-2/AVE52/ANV2/Chicken/MH028405
AvAstV-3/DAstV-C-NGB/Duck/FJ434664
AvAstV-3/G2174/Duck/OR924092
AvAstV-3/TAstV-2/Turkey/AF206663
UCAvAstV/XZN01/Bird/MW826371 (unclassified avastrovirus/strain, or isolate/host/GenBank accession number)
UCAvAstV/D11-148.2/HK/White eye/KT946721
UCAvAstV/D11-139/HK/House crow/KT946718
UCAvAstV/G2332/Goose/OR907134
UCAvAstV/G2332/Goose/OR907134
UCAvAstV/Goose AstV-1/Goose/OM264914
UCAvAstV/G059/Chicken/KT886453
UCAvAstV/Abbotsbury/B/2016/Mute swan/MW588063

**Cluster of mamastroviruses:**
MaAstV-1/HuAstV-1/Human/Z25771 (Mamastrovirus-1/strain, or isolate/host/GenBank accession number)
MaAstV-1/HuAstV-8/Human/MK059956
MaAstV-2/FAstV-D1/Cat/KM017741
MaAstV-3/PoAstV-2/Pig/GQ914773
MaAstV-6/MLB1 HK05/Human/HM450380
MaAstV-7/BdAstV-1/Dolphin/FJ890355
MaAstV-8/NI-295/Human/GQ415660
MaAstV-9/Paris/Human/KM401565
MaAstV-10/CHN/2020-1/Ferret/OL444939
MaAstV-12/LD71/Bat/FJ571067
MaAstV-13/MOxAstV-CH18/Musk ox/MK211323
MaAstV-15/LD77/Bat/FJ571066
MaAstV-18/BF2/Bat/MW450837
MaAstV-19/LD38/Bat/FJ571065
UCMaAstV/LS11/Bat/FJ571068 (unclassified mamastrovirus/strain, or isolate/host/GenBank accession number)
UCMaAstV/Dog AstV/Dog/JQ081297
UCMaAstV/TN/2208/2010/Rabbit/JF729316
UCMaAstV/EEGN4/Bovine/OQ686756
**Cluster of unclassified AstVs in fish:**
UCAstV/Japanese halfbeak/MG599881 (unclassified AstV/detected in fish/GenBank accession number)
UCAstV/Banjos banjos/MG599882
UCAstV/Strawberry cod/MG599883
UCAstV/Wenling perciformes/MG599884
**Invertebrate associated AstV-like virus:**
UCAstV/Penaeid shrimp/KX884564 (unclassified AstV/detected in invertebrate/GenBank accession number)
UCAstV/Crayfish/KX884566
